# Overlapping and unique roles played by ROCK1 and 2 in the modulation of coding and long noncoding RNA expression

**DOI:** 10.1186/s12864-019-5715-0

**Published:** 2019-05-22

**Authors:** He-Ming Zhou, Ji-Gang Zhang, Xue Zhang, Guo-Rong Fan, Gao-Lin Liu, Qin Li

**Affiliations:** Department of Clinical Pharmacy, Shanghai General Hospital, School of medicine, Shanghai Jiao Tong University, No.100 Haining Road, Shanghai, 200080 People’s Republic of China

**Keywords:** ROCK, Microarray, DElncRNA, DEmRNA

## Abstract

**Background:**

Our previous study described the crucial role of Rho-associated coiled-coil containing-kinases (ROCK) in hepatocellular carcinoma (HCC). However, the potential significance of long noncoding RNA downstream of ROCK is largely unknown. Here, a comprehensive comparative bioinformatics analysis of a microarray of an MHCC-97H cell line overexpressing ROCK1 or ROCK2 was performed.

**Results:**

Numerous lncRNAs and mRNAs were deregulated by Rho-associated coiled-coil containing kinases 1 and 2. These results were consistent with the qRT-PCR results. Compared with MHCC-97H-Con, which was transfected with a null vector, the GO analysis revealed differentially expressed mRNAs (DEmRNAs) in MHCC-97H-ROCK1 (ROCK1 was overexpressed) enriched in apoptotic cell clearance, the cyclooxygenase pathway and bone trabecula morphogenesis; the DEmRNAs in MHCC-97H-ROCK2 (ROCK2 was overexpressed) were enriched in VEGF production, chemokine-associated signaling pathways, acute inflammatory response and vasoconstriction. Compared with MHCC-97H-ROCK2, the DEmRNAs in MHCC-97H-ROCK1 were involved in the JAK-STAT cascade, the Akt signaling pathway and the activity of several different peptidases. The pathway analysis of ROCK1 and ROCK2 revealed an overlap in the VEGF signaling pathway, ECM-receptor interaction, and adhesion and differences in the PPAR signaling pathway and mismatch repair. The predicted targets of the differentially expressed lncRNA (DElncRNAs) were enriched in the p53 signaling pathway, Jak-STAT signaling pathway, etc.

Several hub DElncRNAs were identified.

**Conclusions:**

ROCK1 and 2 modulate the expression of numerous mRNAs and lncRNAs and may participate in several signaling pathways in HCC. Several hub molecules were identified in the lncRNA-mRNA networks. Our results provide baseline data for ROCK1 and 2 regulation in HCC that might have implications for further research.

**Electronic supplementary material:**

The online version of this article (10.1186/s12864-019-5715-0) contains supplementary material, which is available to authorized users.

## Background

Rho-associated coiled-coil-containing kinases (ROCK) were originally identified as major effectors of RhoA small GTPase [1–4], and two isoforms, i.e., ROCK1 and ROCK2, have been identified [5]. ROCK belong to the AGC family of serine/threonine kinases and play vital roles in cell. Since the identification of ROCK1 and ROCK2, studies have focused on their roles in the regulation of the actin-myosin cytoskeleton [6]; their actin organization regulates the formation of stress fibers and the focal adhesion complex, apoptosis and development, cell proliferation and cytokinesis, thereby modulating the process of migration, metastasis and invasion in carcinoma. More than two decades after their discovery, their fundamental cell biology, function and molecular structure have been extensively studied [7]. Our previous studies explored how ROCK modulated the pathogenesis of hepatocellular carcinoma (HCC) [8, 9]. Nevertheless, the molecular details of these biochemical mechanisms remain unknown. A better understanding of the global molecular changes mediated by ROCK1 and 2 may be helpful for identifying the mechanism of pathophysiology.

Utilizing shRNA technology to knockdown the expression of ROCK1 and 2 has been reported to induce large-scale changes in global miRNA expression [10]. Moreover, several studies have reported that long non coding RNAs (lncRNAs) may regulate the cytoskeleton by ROCK signaling during tumor migration and metastasis, indicating that ROCK are associated with lncRNA during key steps in carcinoma [11–13]. Similar to its broad effects on global miRNA transcriptional expression, these studies have suggested that the activity of ROCK1 and 2 may affect the level of several coding and long noncoding RNAs. To further understand the functional significance of mRNAs and lncRNAs in ROCK-mediated pathological processes in HCC, RNA sequencing technology was utilized to profile the mRNA and lncRNA expressional signatures in three pairs of ROCK1 overexpression, ROCK2 overexpression and normal control MHCC-97H cell lines, which have a high metastatic ability [14]. Taken together, our study focused on differentially expressed mRNAs (DEmRNAs) and lncRNAs (DElncRNAs) located downstream of Rho/ROCK pathways and formulated network charts to identify crucial nodal molecules.

## Results

### ROCK isoforms were overexpressed in MHCC-97H

ROCK1 and ROCK2 stably expressing cells were established. To determine whether upregulation was achieved, a western blot analysis of whole cell lysates from these experimental samples was conducted to assess the expression levels of ROCK1 and 2. As shown in Additional file 1, both ROCK1 and 2 were observed at detectable levels.

### Overview of the expression profiles

**Fig. 1:**
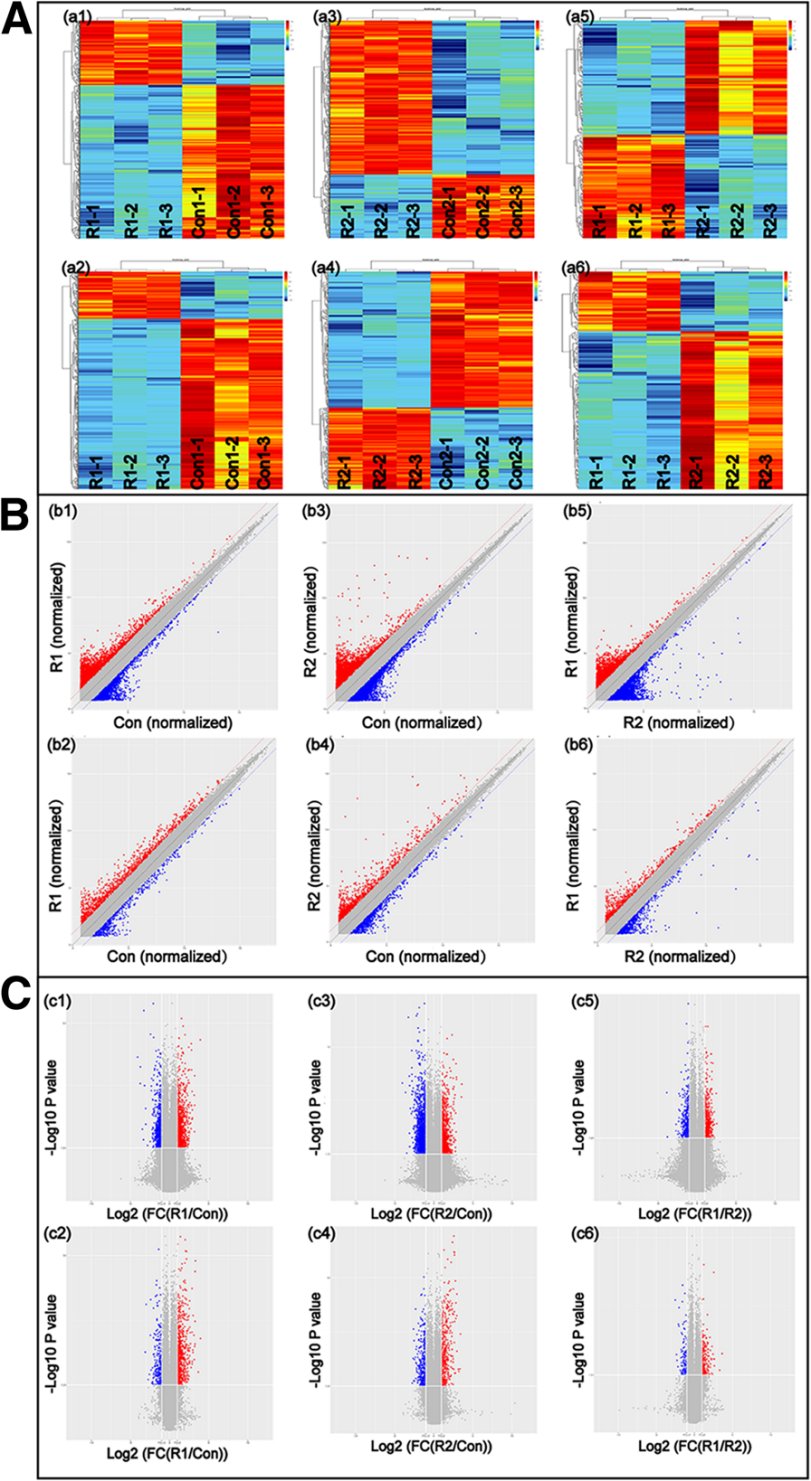
Overview of the microarray data. a **Heatmaps** Heatmaps of DElncRNAs **(a1) and (a3)** and DEmRNAs **(a2) and (a4)** in R1 and R2 compared with Con and in a comparison between R1 and R2 **(a5) and (a6)** are shown. Red indicates higher expression, while green indicates lower expression. b **Scatter plots** Scatter plots of differentially expressed lncRNAs **(b1) and (b3)** and mRNAs **(b2 and b4)** in R1 and R2 compared with Con. DElncRNAs **(b5)** and DEmRNAs **(b6)** in R1 compared with those in R2. The X-axis of the scatter plot represents the normalized signal values from the control group, and the Y-axis of the scatter plot represents the normalized signal values from the test group. The plots falling on the Y = X line (the median line in the figure) represent no signal value differences between the two chips. The plots falling outside of the 45° line on both sides of the median line represent signal value differences between the two chips with a fold change > 2 or < 0.5. The scatter plots show the genes that were upregulated and downregulated by 2-fold with red and green plots, respectively. c **Volcano plots** Volcano plots of DElncRNAs **(c1) and (c3)** and DEmRNAs **(c2) and (c4)** in R1 and R2 compared to Con. DElncRNAs **(c5)** and DEmRNAs **(c6)** in R1 compared with R2. The volcano plots were constructed using the fold change values and *p*-values. The X axis represents Log2 (fold change), and the Y axis represents -log10 (*p*-value). The vertical lines correspond to a 2.0-fold increased and decreased change, and the horizontal line represents a *p-*value = 0.05. The red and green dots represent the upregulated and downregulated genes, respectively

To determine the differences in the mRNA and lncRNA expression profiles among the MHCC-97H-ROCK1, MHCC-97H-ROCK2 and MHCC-97H-Con cell lines, 9 plates of cells were prepared. The total RNAs were extracted for microarray. Using a method of Limma and level of significance of *p* < 0.05 and a fold change (FC) > 2, 840 DEmRNAs were identified in the MHCC-97H-ROCK1 cells, including 183 (21.79%) downregulated and 657 (78.21%) upregulated DEmRNAs, and 1022 DElncRNAs were identified in the ROCK1 upregulated samples, including 303 (29.65%) upregulated and 719 (70.35%) downregulated DElncRNAs. Unsupervised hierarchical clustered heat maps of the expression of DElncRNAs and DEmRNAs in the MHCC-97H-ROCK1 cells are displayed in Fig. 1a **(a1) and (a2)**. Based on Fig. 1a **(a1) and (a2)**, the DElncRNAs and DEmRNAs were all clustered into the MHCC-97H-ROCK1 cells and MHCC-97H-Con cells, respectively. Although the sample size was limited, we found that the DElncRNAs and DEmRNAs could distinguish the MHCC-97H-ROCK1 group from the normal control group. The microarray data of the DElncRNAs and DEmRNAs are presented in scatter plots in Fig. 1b **(b1) and (b2)** and volcano plots in Fig. 1c **(c1) and (c2)**. Among the DElncRNAs, ENST00000568776 and lnc-EXT1–1:1 were the most upregulated and downregulated, respectively. Additionally, NM_000090 (COL3A1) and NM_0012830569 (CCDC198) were the most significantly upregulated and downregulated mRNAs in MHCC-97H-ROCK1, respectively. All DElncRNAs were distributed in all chromosomes, including sex chromosomes X and Y. Furthermore, the DEmRNAs were widely distributed in chromosomes other than sex chromosome Y.

Subsequently, using the same level of significance of *p* < 0.05 and FC > 2, 1119 DElncRNAs were observed between the MHCC-97H-ROCK2 group and normal control group, including 327 (29.22%) DElncRNAs that were more expressed than usual and 792 (70.78%) DElncRNAs that were less expressed than usual. ENST00000568776 was the most upregulated, while lnc-UCK1–2:1 was the most downregulated among all DElncRNAs. In total, 500 DEmRNAs were filtered, including 312 (62.40%) upregulated DEmRNAs, and the remaining (37.60%) DEmRNAs were downregulated. NM_005780 (LHFPL6) was the most upregulated DEmRNA, with an FC value of 10.76. NM_001004686 (OR2 L2) was the most downregulated DEmRNA, with an FC value of 6.70. The features of the DElncRNAs and DEmRNAs are illustrated in a heatmap **(**Fig. 1a **(a3) and (a4))**, scatter plots **(**Fig. 1b **(b3) and (b4))** and volcano plots **(**Fig. 1c **(c3) and (c4))**. The DElncRNAs were ubiquitously expressed in all chromosomes from chromosome 1 to chromosome Y. The DEmRNAs were also extensively distributed. The DEmRNAs were distributed in all chromosomes except for sex chromosome Y.

Next, we compared the expression characteristics of the following two isoforms: ROCK1 and ROCK2. The screening criteria were set at *p* < 0.05 and FC > 2, and the results showed that the number of total DElncRNAs was 174. Compared with the MHCC-97H-ROCK2 cells, the expression of 83 (47.70%) lncRNAs was decreased, and the expression of 91 (52.30%) lncRNAs was increased in the MHCC-97H-ROCK1 cells. Similarly, we detected 197 DEmRNAs between the MHCC-97H-ROCK1 group and the MHCC-97H-ROCK2 group, including 54 (27.41%) DEmRNAs that were lowly expressed and 143 (72.59%) DEmRNAs that were highly expressed. Heatmaps **(**Fig. 1a **(a5) and (a6))**, scatter plots **(**Fig. 1b **(b5) and (b6))** and volcano plots **(**Fig. 1c **(c5) and (c6))** were intuitively constructed. NM_194286 (SRRM4) and NM_021957 (GYS2) were the most up- and downregulated DEmRNAs. In this comparison, the DEmRNAs were distributed in all euchromosomes and sex chromosome X but not in sex chromosome Y. However, the DElncRNAs were not distributed in euchromosomes 21 and 22 and sex chromosome Y. Taken together, these results highlight the specificity and distinction of the ROCK1 and ROCK2 isoforms. We have uploaded our data files according to the requirement of GEO and our records have been approved and assigned GEO accession numbers by GSE122145.

### GO, KEGG and Reactome analysis of differentially expressed mRNAs

The functional implication of the DEmRNAs was further investigated by GO [15], KEGG [16] and Reactome [17] enrichment analyses. Based on the GO analysis, the DEmRNAs were associated with molecular function, biological process and cellular components.

**Fig. 2:**
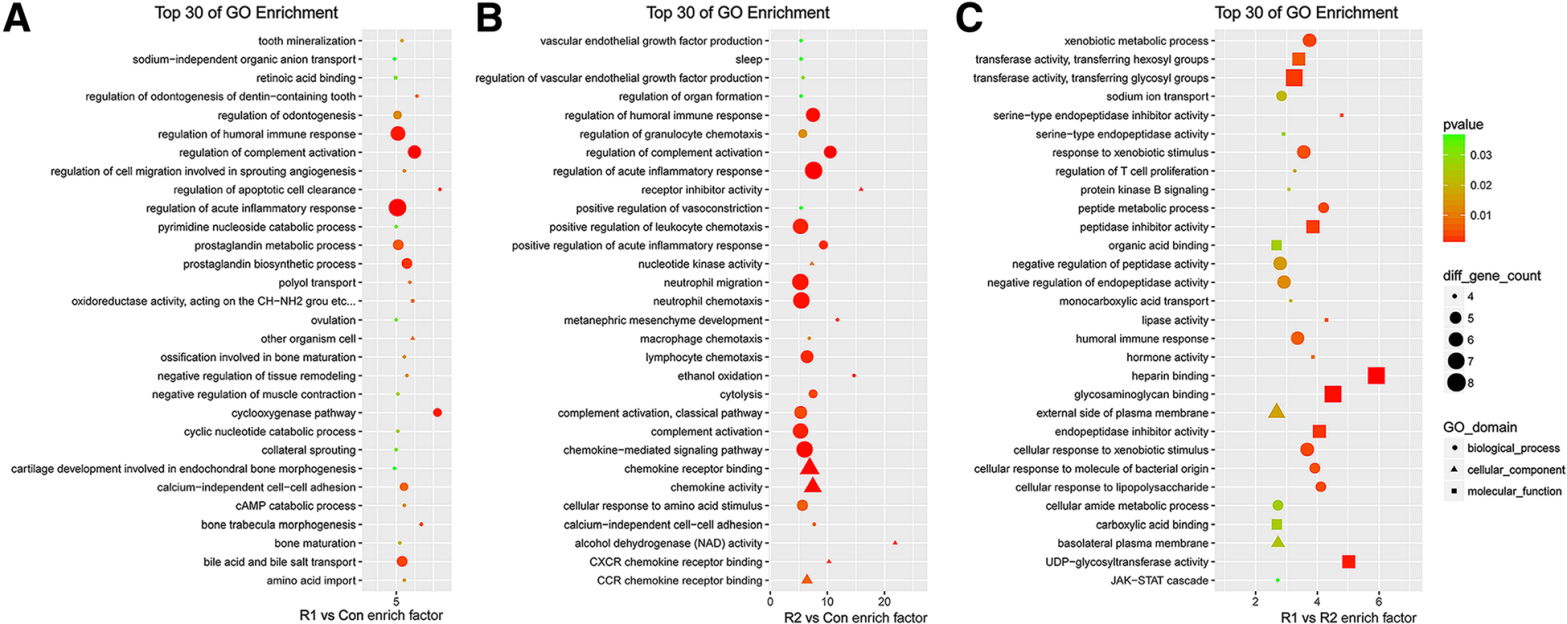
**GO enrichment analysis of DEmRNAs.** The top 30 GO enrichment terms of the DEmRNAs are listed. The left terms (**a**) represent the R1 vs Con comparison, the middle terms (**b**) represent the R2 vs Con comparison and the right terms (**c**) represent the R1 vs R2 comparison. The bubble charts illustrate the terms in which the DEmRNAs were enriched. The enrichment factor was calculated by (the number of different genes in a term/total number of different genes in a term)/(total number of genes in a term/total number of genes in a database). A *p*-value < 0.05 was considered statistically significant

First, compared with the MHCC-97H-Con cells, the GO enrichment analysis (Fig. 2a) showed that in the MHCC-97H-ROCK1 cells, the DEmRNAs were especially enriched in following subcategories: regulation of apoptotic cell clearance, the cyclooxygenase pathway and bone trabecula morphogenesis. Calcium independent cell-cell adhesion and the regulation of cell migration, which are involved in sprouting angiogenesis, were also included in the top 30 GO terms. These results indicate that these DEmRNAs, which were specifically expressed in the MHCC-97H-ROCK1 cells compared with those in the MHCC-97H-Con cells, possibly participate in reactive oxygen species (ROS)-mediated cell apoptosis or cause modality change.

Therefore, we identified DEmRNAs between the MHCC-97H-ROCK2 group and the control group. Based on the GO enrichment analysis (Fig. 2b), the DEmRNAs were implicated in vascular endothelial growth factor (VEGF) production, several chemokine-associated signaling pathways and the acute inflammatory response. Regulation vasoconstriction was also included in the top 30 GO terms. Moreover, we contrasted the MHCC-97H-ROCK1 group with the MHCC-97H-ROCK2 group. The GO enrichment analysis (Fig. 2c) suggested that the DEmRNAs were involved in the JAK-STAT cascade, protein kinase B (Akt) signaling pathway, and the activity of several different peptidases. We could likely delve deeper into these terms to identify more distinctive factors between ROCK1 and ROCK2.

**Fig. 3:**
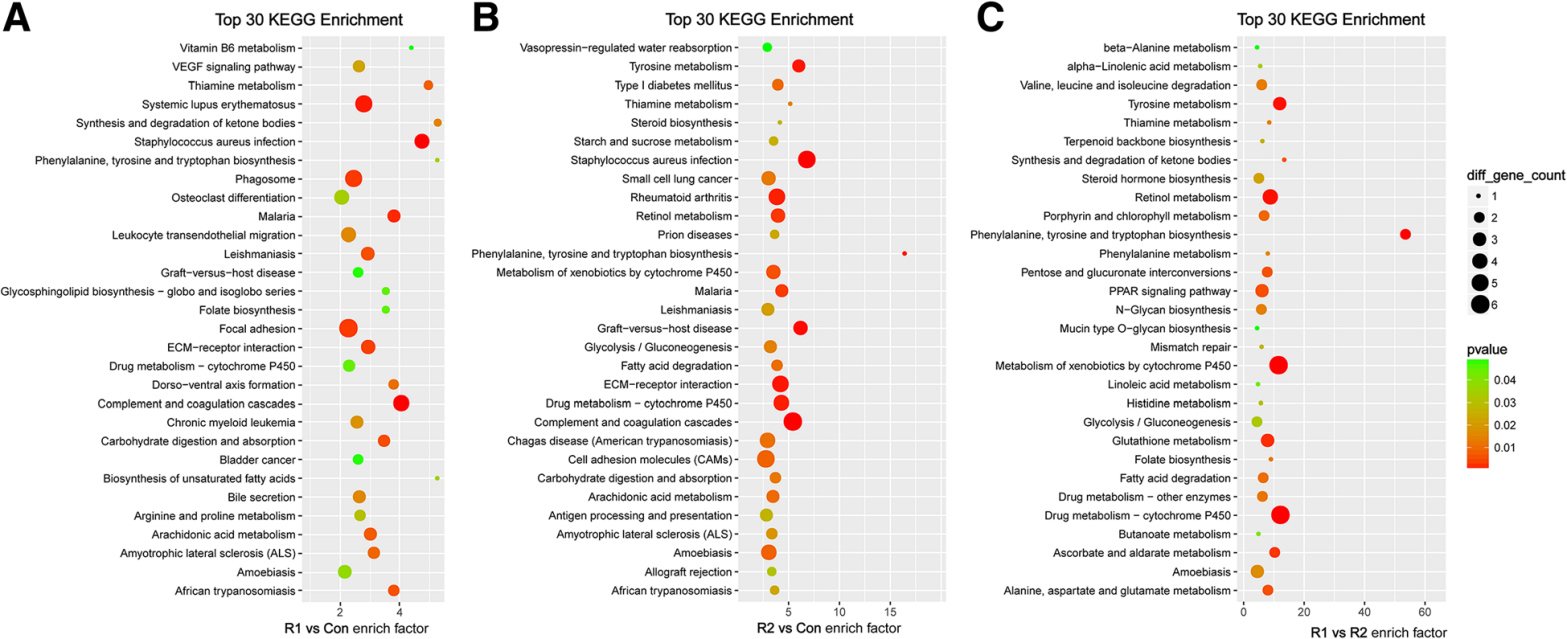
Pathway enrichment analysis of DElncRNAs. The top 30 pathway enrichment terms of the DElncRNAs are listed. **a** represents the R1 vs Con comparison, **b** represents the R2 vs Con comparison and (**c**) represents the R1 vs R2 comparison. The bubble charts illustrate the terms in which the DElncRNAs were enriched. The enrichment factor was calculated by (the number of different genes in a term/total number of different genes in a term)/(total number of genes in a term/total number of genes in a database). *P* < 0.05 was considered statistically significant

A KEGG analysis was performed to identify the possible pathways of ROCK1 and 2. Compared with the MHCC-97H-Con cells, the DEmRNAs in the ROCK1 upregulated groups were enriched in the VEGF signaling pathway, ECM-receptor interaction and focal adhesion (Fig. 3a). Compared with the normal control group, extracellular matrix (ECM)-receptor interaction and cell adhesion molecules (CAMs) participated in the ROCK2-mediated biological processes (Fig. 3b). In addition, diseases, such as Graft-versus-host disease, malaria and cancer, were enriched in both comparison groups, suggesting that the two isoforms were both a part of the parameters that induced these pathogeneses. Furthermore, the MHCC-97H-ROCK1 and MHCC-97H-ROCK2 cells were contrasted (Fig. 3c); the DEmRNAs were clustered in the peroxisome proliferator-activated receptor (PPAR) signaling pathway, mismatch repair and metabolism of many substances, suggesting the distinctive functions of the isoforms.

Reactome analysis was performed for further pathway enrichment. Detailed information in Supporting Materials. To sum up, comparing with MHCC-97H-Con, the DEmRNAs in the MHCC-97H-ROCK1 affected cell migration, assembly and organization of ECM, p53 signaling pathway which regulated apoptosis and senescence, regulation of gene expression etc., which was consistent with GO and KEGG analysis; the DEmRNAs in the MHCC-97H-ROCK2 clustered in inflammatory response, which was similar to GO and KEGG analysis. Moreover, the MHCC-97H-ROCK1 and MHCC-97H-ROCK2 cells were contrasted, with Reactome showing that DEmRNAs focused on metabolic disorder. Specially, the results of Reactome emphasized DEmRNAs in this comparison involved in several interleukin-associated signaling pathways, which distinguished with GO and KEGG.

### GO, KEGG and Reactome enrichment analyses of DElncRNA prediction targets

**Fig. 4:**
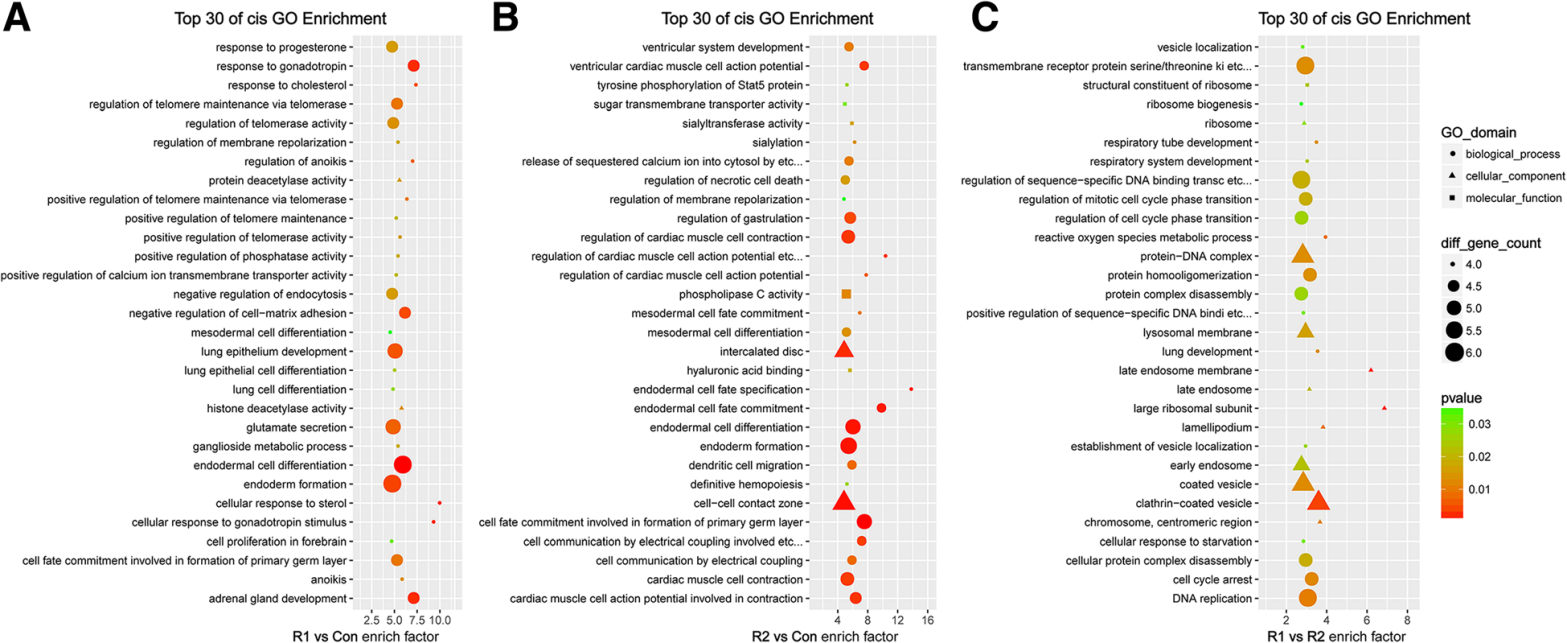
GO enrichment analysis of cis prediction targets. The top 30 GO enrichment terms of the cis prediction target genes of the DElncRNAs are listed. **a** represents the R1 vs Con comparison, **b** represents the R2 vs Con comparison and (**c**) represents the R1 vs R2 comparison. The bubble charts illustrate the GO terms in which the cis target genes of the DElncRNAs were enriched. The enrichment factor was calculated by (the number of different genes in a term/total number of different genes in a term)/(total number of genes in a term/total number of genes in a database). A *p*-value< 0.05 was considered statistically significant

**Fig. 5:**
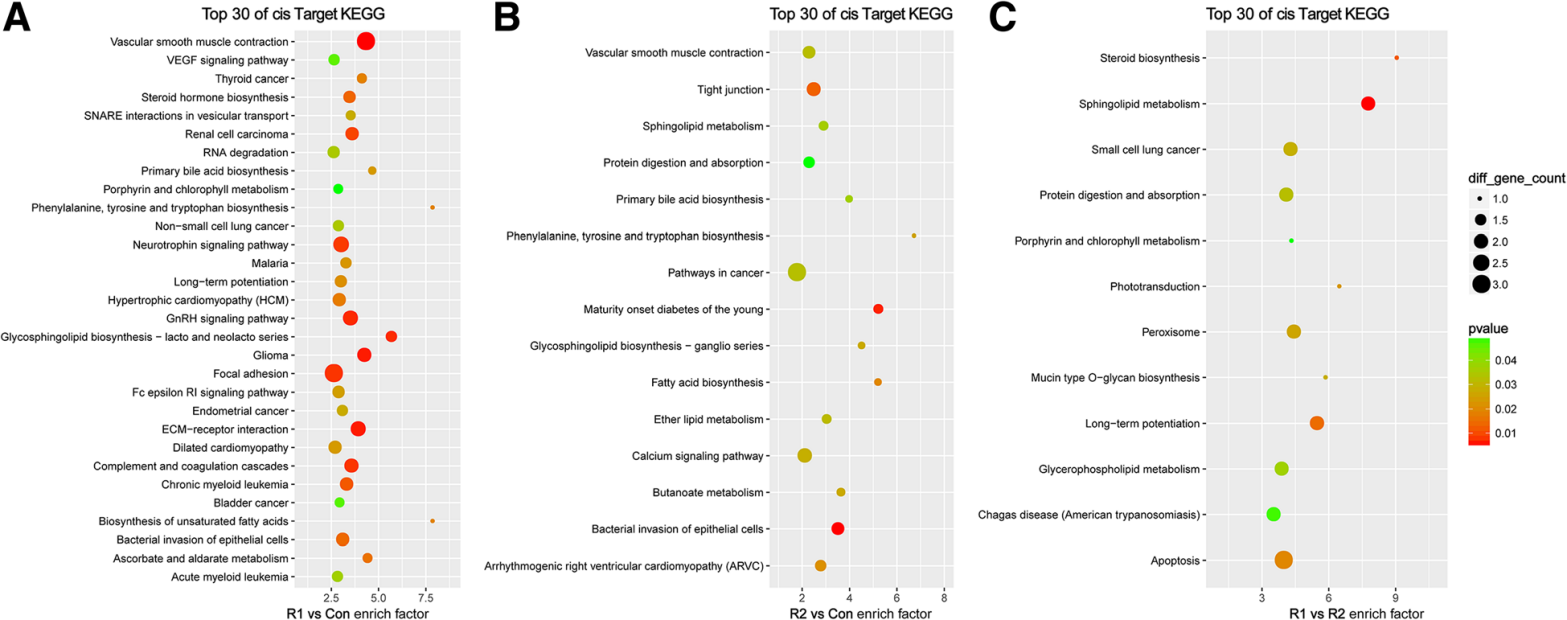
KEGG analysis of cis prediction targets. The top 30 KEGG enrichment terms of the cis prediction target genes of the DElncRNAs are listed. **a** represents the R1 vs Con comparison, **b** represents the R2 vs Con comparison and (**c**) represents the R1 vs R2 comparison. The bubble charts illustrate the KEGG terms in which the cis target genes of the DElncRNAs were enriched. The enrichment factor was calculated by (the number of different genes in a term/total number of different genes in a term)/(total number of genes in a term/total number of genes in a database). A *p*-value < 0.05 was considered statistically significant

LncRNAs have been shown to modulate the expression of adjacent and distantly located target genes by cis- or trans-acting regulation, respectively. Altogether, 759 cis target genes and 9391 trans target genes were identified in the GO and KEGG analyses. Compared with MHCC-97H-ROCK1 and MHCC-97H-Con, the cis target GO (Fig. 4a) was mainly enriched in cell response to chemicals, cell differentiation and regulation of telomerase, while the trans target GO (Additional file 2)A principally focused on fatty acid-related metabolic processes. Cis target KEGG enrichment (Fig. 5a) mainly clustered in several cancers and carcinomas, vascular smooth muscle contraction and focal adhesion, while the trans target KEGG (Additional file 3)**A** were enriched in the p53 signaling pathway, lysosome and ubiquinone and other terpenoid-quinone biosynthesis. In contrast to MHCC-97H-ROCK2 and MHCC-97H-Con, the cis target GO enrichment (Fig. 4b) focused on the terms endodermal cell fate specification and commitment and regulation of cardiac muscle cell action potential, while the trans target GO (Additional file 2)**B** mainly clustered in antigen processing, presentation of exogenous processing, T cell mediated cytotoxicity and MHC class I protein complex, all of which are involved in the immune system. The Cis target KEGG (Fig. 5b) enrichment was mainly observed in pathways in cancer and the calcium signaling pathway, while the trans target KEGG (Additional file 3) **B** enrichment was observed in the p53 signaling pathway and Jak-STAT signaling pathway. Compared with MHCC-97H-ROCK2, 132 cis target genes and 1891 trans target genes were identified in MHCC-97H-ROCK1 and subjected to GO and KEGG analyses. The DElncRNA target prediction in MHCC-97H-ROCK1 indicated the following: the cis target GO (Fig. 4c) was mainly enriched in ribosome, endosome and vesicular transport, while the trans target GO (Additional file 2) **C** principally focused on satellite cell proliferation, pyramidal neuron development and differentiation. The Cis target KEGG (Fig. 5c) enrichment mainly focused on fatty acid metabolism, while the trans target KEGG (Additional file 3) **C** enrichment was observed in the p53 signaling pathway, ubiquinone and other terpenoid−quinone biosynthesis and substance metabolism.

To verify the results of GO and KEGG, Reactome analysis was performed for more thorough functional prediction. Detailed information in Additional file 4. Comparing with MHCC-97H-Con, the DElncRNAs in the MHCC-97H-ROCK1 mainly affected immune response, apoptosis and proliferation and cancer; the DElncRNAs in the MHCC-97H-ROCK2 clustered in endosomal/vacuolar pathway, antigen processing, p53 signaling pathway, STAT signaling and so on. Moreover, the MHCC-97H-ROCK1 and MHCC-97H-ROCK2 cells were contrasted, with Reactome showing that DElncRNAs focused on immune response, cell death and survival, RNA editing, DNA repair, p53 signaling pathway and so on. Generally, the results of Reactome and GO & KEGG were highly coincident, several aspects will be the focus of further investigation.

### Analysis of lncRNA-mRNA interaction network

**Fig. 6:**
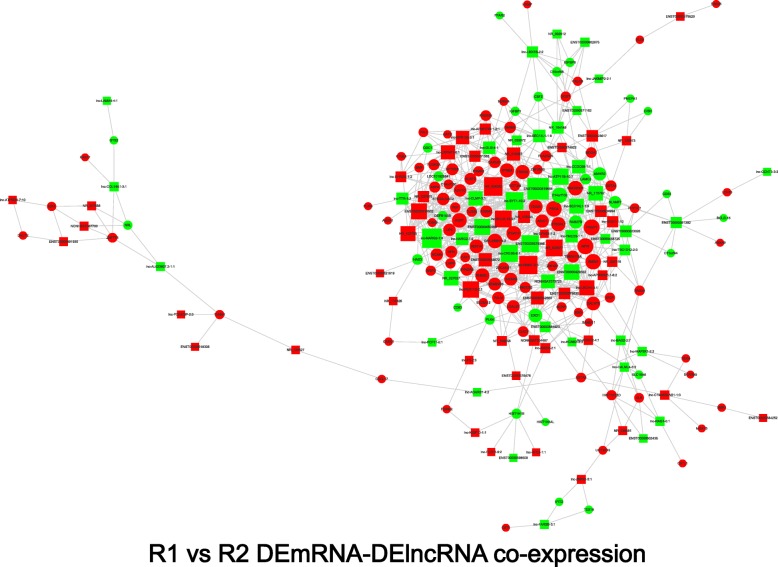
Coexpression network of R1 vs R2. The DElncRNA-DEmRNA coexpression network of R1 vs R2. Rectangle nodes represent lncRNAs, and circular nodes represent mRNAs. The lines between the nodes represent interactions between two genes. Red nodes indicate upregulated genes, while green nodes indicate downregulated genes. The degree was determined by the number of links one node has with other nodes

Given that the interaction between mRNAs and lncRNAs downstream of ROCK1 and 2 has not been well studied. LncRNA-mRNA coexpression networks were constructed to determine the roles of lncRNAs according to their coexpressed mRNAs. Using the criteria of a Pearson’s r ≥ 0.9985 and *p*-value ≤0.0001, the coexpression network consisted of 781 pairs of DElncRNAs and DEmRNAs between the MHCC-97H-Con and MHCC-97H-ROCK1 cells. In the interaction network (Additional file 5), 498 pairs presented as positive, and the remaining pairs presented as negative. In total, 174 DElncRNAs (62 downregulated and 112 upregulated), and 231 mRNAs (33 downregulated and 198 upregulated) were included. ENST00000579529, lnc-BTF3–4:1 and ENST00000445272 were the top 3 lncRNA omphalos, while FBXO2, HIST1H3F and ODC1 were the hub mRNAs in the network. The lncRNA-mRNA pairs ENST00000598518-SYTL2, lnc-MRGPRF-6:1-C1R and ENST00000505709-CREBFR were the most positively related, which may indicate that these lncRNAs could elevate the expression of these relevant mRNAs. In contrast, ENST00000524346-RNASE1, lnc-SMEK2–1:1-TTC23 and lnc-NARG2–1:4-CX3CL1 were the top 3 negatively expressed pairs, suggesting that these lncRNAs are likely to inhibit the expression of the related mRNAs. An interaction network containing coding and noncoding genes was constructed using the same inclusion criteria between the MHCC-97H-Con and MHCC-97H-ROCK2 cells (Additional file 6). This network contained 396 pairs of DElncRNAs and DEmRNAs, including 190 pairs that were positively coexpressed and 206 pairs that were negatively coexpressed. Lnc-TSPY10–2:1, lnc-MPHOSPH8–3:1 and NR_028366 were the top 3 lncRNAs that interacted with other genes in a great degree, while DPYSL3, MPP1 and GPRC5B were the top 3 hub mRNAs that mediated the most connections with other molecules. The lncRNA-mRNA pairs with the highest positive correlation coefficients included NR_125925-TNFRSF1B, lnc-SSX1–5:1-SSX3 and ENST00000508884-ADTRP. The pairs NR_120619-GPRC5B, lnc-MPHOSPH8–3:1-HACD1 and lnc-TSPAN11–2:8-TMEM98 had the highest negative correlation coefficients. The coexpression network between MHCC-97H-ROCK1 and MHCC-97H-ROCK2 (Fig. 6) consisted of 824 connections with 95 DElncRNAs and 120 coding genes. In this network, 419 pairs presented as negative, and 405 pairs presented as positive. When the degree of the connection in the network was calculated, ENST00000619654, lnc-NARG2–1:4, NR_024042 and lnc-RNMT-2:5 were identified as playing central roles in the regulation process. CARNS1 and lnc-GPR135–3:1, GS1–259H13.2 and lnc-PEX11B-2:1, and LAMC2 and lnc-CCDC68–1:1 were the most positively associated pairs, while ASIC1 and ENST00000579386, TMEM229B and lnc-SYT1–10:2, and MACROD1 and lnc-SEC14 L1–1:9 were the most negatively associated pairs.

### qRT-PCR verification

**Fig. 7:**
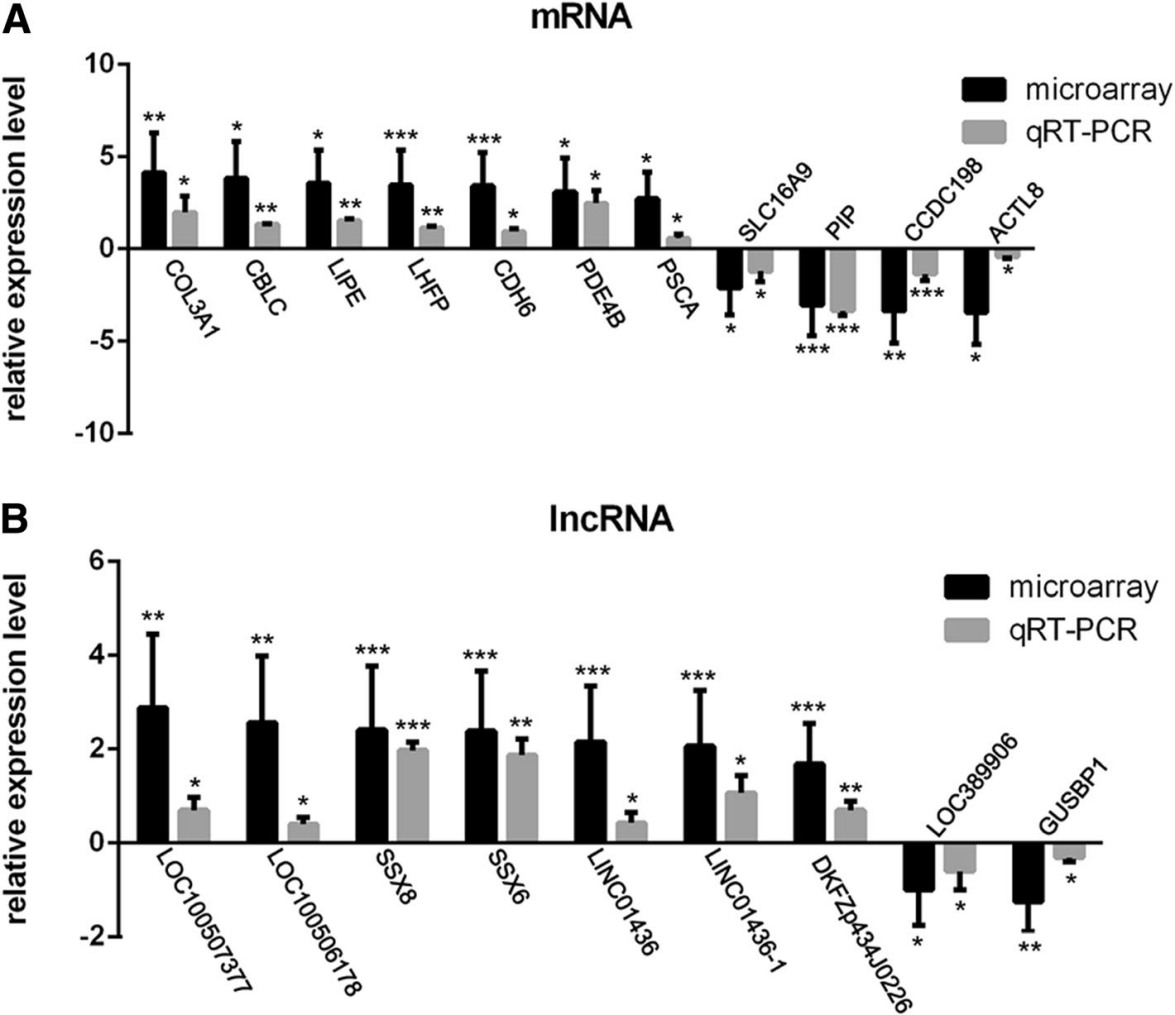
QRT-PCR verification. Validation of DEmRNAs (**a**) and DElncRNAs (**b**) by qRT-PCR. Comparison of relative expression was calculated by the log2 (fold change) equation. *p < 0.05, **p < 0.01, and ***p < 0.001

The verification of the microarray data was performed using 11 mRNAs and 9 lncRNAs that were possibly involved in ROCK signaling according to the bioinformatics analysis. Effect size (cohen’s d) of t-test analysis from microarray was listed in the brackets following each verified gene. The results of the qRT-PCR analysis (Fig. 7) revealed that the specific mRNAs COL3A1 (effect size: 16.04), LHFP (effect size: 15.06), LIPE (effect size: 3.50), PSCA (effect size: 4.64), CBLC (effect size: 5.30), CDH6 (effect size: 24.71), and PDE4B (effect size: 4.54) and lncRNAs SSX6 (effect size: 29.39), SSX8 (effect size: 15.75), LOC100507377 (effect size: 6.93), LOC100506178 (effect size: 15.75), LINC01436 (effect size: 9.96), LINC01436–1 (effect size: 14.01), and DKFZp434J0226 (effect size: 8.14) had increased steady-state expression levels, whereas the mRNAs CCDC198, ACTL8, PIP, and SLC16A9 and lncRNAs GUSBP1 and LOC389906 had decreased steady-state expression levels relative their matched counterparts. The qRT-PCR analysis indicated that our bioinformatics methods and analyses were dependable.

## Discussion

The deregulation of Rho family small guanosine triphosphatases has been implicated in human carcinogenesis. Rho-kinases are direct downstream effectors of Rho guanosine triphosphatases, which participate in the regulation of cytoskeletal reorganization and motility. Nevertheless, previous studies have utilized nonselective inhibitors of ROCK1 and 2 kinase activity and promiscuously inhibited both isoforms. Here, we analyzed the specific function of ROCK1 and 2 in HCC with a complete set of informatic tools. DEmRNAs and DElncRNAs that met the screening criteria were identified, and many unknown biological functions likely exist among such a massive amount of data.

Previously, published studies have reported that the shRNA-knockdown of ROCK1 and 2 and Y27632 treatment induces large-scale changes in messenger RNA (mRNA) and microRNA (miRNA) expression [10, 18]. However, the regulation of lncRNAs by ROCK1 and 2 is unknown. Considering that ROCK are major regulators of migration and invasion [19], our findings unsurprisingly revealed enrichment in adhesion, vasoconstriction and the VEGF signaling pathway. Furthermore, several miRNAs have been shown to be involved in proliferation, calcium transport, cell-cycle regulation and neurogenesis [10]. The COL3A1 gene is predicted to be associated with HCC [20, 21]. Moreover, this gene has been shown to be differentially expressed with ROCK alterations [10, 22] and is predicted to modulate the expression of several collagen genes during development [23]. Here, we found the COL3A1 was significantly upregulated in MHCC-97H-ROCK1, revealing its role in HCC progression.

Previous studies have shown that lncRNAs participate in epigenetic, transcriptional and posttranscriptional regulation [24–26]. The occurrence and development of HCC, including proliferation, adhesion and apoptosis, aligns with the central dogma ‘DNA → RNA → protein’. In addition, with the development of whole genome and transcriptome sequencing technologies, lncRNAs have provided an important new perspective regarding the centrality of RNA in gene regulation. To date, knowledge regarding the function of lncRNAs mediated by ROCK in HCC is lacking. ROCK proteins have been shown to modulate proliferation by affecting lncRNAs, such as lncRNA – Six1 [27] and NONMMUG014387 [28]. However, these regulations have not been illustrated via protein-lncRNA interactions. Thus, these regulations may depend on particular hub molecules, such as ENST00000579529, FBXO2, lnc-TSPY10–2:1 and DPYSL3.

In addition, several scientific studies have reported that ROCK plays a vital role in cancer, including tissue invasion and metastasis [29], sustained angiogenesis [30, 31], apoptosis evasion [32–34], and DNA damage [34]. However, the exact signaling pathways are unclear. By performing GO and KEGG analyses, our research found that the VEGF signaling pathway, chemokine-associated signaling pathway, TGF-beta signaling pathway, mTOR signaling pathway, Wnt signaling pathway, RIG-I-like receptor signaling pathway, MAPK signaling pathway and p53 signaling pathway were involved (some data are not shown). The relationships between p53 and ROCK [35, 36] and between MAPK and ROCK [37, 38] in cancers has received limited attention, while other relevant relationships remain largely unexplored. Therefore, this study could provide valuable clues for further ROCK-mediated pathway analyses at the nucleic acid level in HCC.

Taken together, increasing attention to mRNA and lncRNAs, which define the molecular portraits of ROCK-mediated signaling pathways, and the identification of the deeper molecular mechanism of the biological progression of HCC could undoubtedly enhance our comprehension of both the ROCK isoforms and HCC.

## Conclusion

ROCK1 and 2 modulated the expression of numerous mRNAs and lncRNAs and may participate in several signaling pathways in HCC. Several hub molecules in lncRNA-mRNA networks were identified. Our results provide baseline data regarding ROCK1 and 2 regulation in HCC that might have implications for further research.

## Methods

### Establishment of ROCK1 and ROCK2 stably expressing cells

The human liver cancer cell line MHCC-97H was obtained from Cell Bank of Chinese Academy of Sciences (Shanghai, China). The cell catalog number is SCSP-528. One day before transfection, 2 × 10^4^ cells were seeded onto a 24-well plate. A lenti-virus (Obio, Shanghai, China) was used to transduce the cells with the corresponding vectors. After 24 h, the transduced cells were diluted 1:100 and plated onto a 100-mm culture dish. To select the stable transformants, the cells were cultured in high-glucose Dulbecco’s modified Eagle’s medium (DMEM; Gibco, Carlsbad, CA, USA) with 5 μg/mL polybrene for 2 weeks. Clones displaying polybrene resistance and expressing the fluorescent label (GFP) were selected and expanded. The stable overexpression of ROCK1 and ROCK2 was confirmed by western blotting.

For the lentivirus construction, the full length of ROCK1 was cloned into the overexpression vector Lenti-CMV-MCS-3FLAG-PGK-Puro H156 to produce pLenti-CMV- ROCK1-3FLAG-PGK-Puro (Obio Technology Co., Ltd., Shanghai, China). ROCK1 was amplified using the following primer set: 5′- CGCAAATGGGCGGTAGGCGTG -3′ and 5′- CAGCGGGGCTGCTAAAGCGCATGC -3′. Then, the full length of ROCK2 was cloned into the over-expression vector pLenti-CMV-MCS-3FLAG-PGK-Puro H156 to produce pLenti-CMV-ROCK2-3FLAG-PGK-Puro (Obio Technology Co., Ltd., Shanghai, China). ROCK2 was amplified using the following primer set: 5′- CGCAAATGGGCGGTAGGCGTG -3′ and 5′- CAGCGGGGCTGCTAAAGCGCATGC -3′.

### Cell culture and treatment

Stable cell lines of MHCC-97H-Con, MHCC-97H-ROCK1, and MHCC-97H-ROCK2 were established by Obi Technology (Shanghai) Corp., Ltd. The above cell lines were maintained in DMEM supplemented with 10% fetal bovine serum (FBS; Gibco), 100 μg/mL streptomycin, 100 U/mL penicillin (Gibco) and 2 μg/mL puromycin (InvivoGen) at 37 °C with 5% CO_2_. The cells were treated with DMEM for 24 h, and then, the total RNA was extracted and used for the lncRNA and mRNA microarray, RT-PCR and qRT-PCR.

### RNA isolation

TRIzol Reagent (Cat# 15596–018, Life Technologies, Carlsbad, CA, US) was applied to extract the total RNA following the manual, and the RIN was evaluated to confirm the RNA integrity using an Agilent Bioanalyzer 2100 (Agilent Technologies, Santa Clara, CA, US). Eligible total RNA, which was defined as RIN ≥ 7.0, 28S/18S ≥ 0.7, was further purified using RNeasy Mini Kit (Cat# 74106, QIAGEN GmBH, Germany) and RNase-Free DNase Set (Cat# 79254, QIAGEN GmBH, Germany).

### RNA microarray

The sample preparation and microarray hybridization were performed by SBC Biotech, Shanghai, People’s Republic of China. In short, a Low Input Quick Amp Labeling Kit One-Color (Cat.# 5190–2305, Agilent Technologies, Santa Clara, CA, US) was used for the total RNA amplification and labeling according to the protocol. The labeled complementary RNA (cRNA) was then purified by an RNeasy Mini Kit (Cat.# 74,106, QIAGEN GmBH, Germany). 1.65 μg Cy3-labeled cRNA was used to hybridize each slide by a Gene Expression Hybridization Kit (Cat.# 5188–5242, Agilent Technologies, Santa Clara, CA, US) in a hybridization oven (Cat.# G2545A, Agilent Technologies, Santa Clara, CA, US) following the operation guide. After 17 h of hybridization, the slides were washed in staining dishes (Cat.# 121, Thermo Shandon, Waltham, MA, US) by a Gene Expression Wash Buffer Kit (Cat.# 5188–5327, Agilent Technologies, Santa Clara, CA, US) following the manufacturer’s instructions. An Agilent Microarray Scanner (Cat# G2565CA, Agilent Technologies, Santa Clara, CA, US) was used for slides scanning. The default settings were as followed: dye channel: Green, Scan resolution = 3 μm, PMT 100%, and 20 bit. The type of Agilent array used was Agilent lncRNA array (Agilent design ID: 074348). The data were extracted with Feature Extraction software 10.7 (Agilent Technologies, Santa Clara, CA, US). The raw data were normalized using the Quantile algorithm in the Limma package in R.

### Differential expression analysis

The mRNAs and lncRNAs reported as being differentially expressed possessed a *p*-value < 0.05 (Student’s *t*-test) with a change at least two-fold above or below the antithetic samples. To obtain a visual overview of the characteristics of the lncRNA and mRNA expression profiles, heat maps, scatter plots, and volcano plots were generated based on the normalized values of the DElncRNAs and DEmRNAs provided by the R package.

### Prediction of DElncRNA targets

The lncRNA target prediction was performed by assessing the cis/trans-regulatory effects. The DElncRNAs and their potential target genes were matched by UCSC gene annotations (http:// genome.ucsc.edu/). The prediction method for the cis regulation target genes was selecting the genes within 10 kb of the lncRNA [39]. The trans regulatory target gene prediction method was conducted using the gene sequence of the corresponding species in the database and selecting the sequences that are complementary or similar through BLAST. Then, RNAplex was applied to calculate the complementary energy between two sequences, and the genes with e < − 30 were selected as trans targets.

### Gene ontology (GO), KEGG and Reactome pathway analysis

The identified DEmRNAs and predicted target genes of the DElncRNAs in the expression profile were assessed via GO and pathway analyses. Shanghai Biotechnology Corporation carried out the analysis. Fisher’s extract test was performed in the clusterProfiler package in R/Bioconductor. The SAA pathway enrichment suite (Shanghai Biotechnology Corporation) was applied to conduct the pathway enrichment analysis. A false discovery rate of *p* < 0.05 was set as the cutoff for both significantly enriched functional GO and Kyoto Encyclopedia of Genes and Genomes (KEGG) pathways. Since GO gives a bit vague classification, another enrichment tool Reactome was used for further verification of results. Detailed information in Supporting Materials.

### Construction of the lncRNA-mRNA coexpression networks

LncRNA-mRNA coexpression networks were constructed based on the correlations between the DEmRNAs and DElncRNAs. For each lncRNA-mRNA pair, Pearson correlation coefficients were calculated. Pearson’s r ≥ 0.9985 and *p*-values ≤0.0001 were selected as the inclusion criteria, and the plots were generated by Cytoscape [40]. In the network diagrams, the rectangle nodes represent the lncRNAs, while circular nodes represent the mRNAs. Upregulation is shown in red, and downregulation is shown in green. The size of the nodes represents the number of related molecules; the larger the nodes, the more the molecules were related.

### Cross-validation by RT-PCR (quantitative real-time PCR)

RNA was reverse transcribed into complementary DNA (cDNA) by a Takara reverse transcription kit (Takara, Kusatsu, Japan) following the manufacturer’s recommendations. The cDNA amplification was performed using TB Green Premix Ex Taq II (Takara, Kusatsu, Japan) on a 7900HT Sequence Detection System (ABI, Foster City, CA, USA). The relative expression of the mRNAs and lncRNAs was calculated by the 2^−ΔΔCt^ method, and GAPDH was used as the internal reference gene. The expression differences were calculated by paired *t-*test. Statistical significance was considered to occur at a *p*-value < 0.05 (*p < 0.05; **p < 0.01; and ***p < 0.001). All reactions were performed in triplicate.

### Western blot analysis

Whole cell protein extracts were prepared according to the method described by Zhang et al. [8]. Polyvinylidene difluoride (PVDF) membranes were incubated with the primary anti-Flag (1:500; Sigma) and anti-GAPDH (1:1000; Abcam) antibodies and a secondary antibody conjugated to HRP (1:50000, Sigma-Aldrich). The respective proteins were detected using a Clarity™ Western ECL Blotting Substrate (BioRad) and a G:BOX imaging system (Syngene, Cambridge, UK) according to the manufacturer’s instructions.

### Data analysis

The data are expressed as the mean ± SD of three independent experiments with three biological replicates. The statistical analysis was performed using SPSS V.18.0 Software (SPSS Inc., Chicago, IL, USA). Based on the fold change, Student’s *t*-test and paired *t*-test, we analyzed the statistical significance of the microarray and RT-PCR results. Statistical significance was considered to occur at *p*-values < 0.05 (two-tailed).

## Additional files

Additional file 1: Establishment of ROCK1 & 2 stably expressing cells. Western blot was performed to confirm the protein expression of ROCK1 & 2 in MHCC-97H cell lines, using GAPDH as internal reference. Left to right – MHCC-97H (97H), MHCC-97H-Con (Con), MHCC-97H-ROCK1 (R1) and MHCC-97H-ROCK2 (R2). (TIF 3040 kb)

Additional file 2: GO enrichment analysis of trans prediction target. The top30 GO enrichment terms of trans prediction target genes of DElncRNAs were listed. The left one represented R1 vs Con, the next represented R2 vs Con and the last one represented R1 vs R2. The bubble charts illustrated the GO terms in which trans target genes of DElncRNAs enriched. The enrich factor was calculated by (number of different genes in a term/total number of different genes in a term) /(total number of genes in a term/total number of genes in a database). *P* < 0.05 was considered statistically significant. (TIF 6686 kb)

Additional file 3: KEGG analysis of trans prediction target. The top30 pathway enrichment terms of trans prediction target genes of DElncRNAs were listed. The left one represented R1 vs Con, the next represented R2 vs Con and the third one represented R1 vs R2. The bubble charts illustrated the KEGG terms in which trans target genes of DElncRNAs enriched. The enrich factor was calculated by (number of different genes in a term/total number of different genes in a term) /(total number of genes in a term/total number of genes in a database). *P* < 0.05 was considered statistically significant. (TIF 5262 kb)

Additional file 4: Method and Results of Reactome pathway analysis. The main functional implication of the DEmRNAs and DElncRNAs by Reactome analysis was listed, which included comparison between 3 groups: R1 vs Con, R2 vs Con and R1 vs R2. (DOCX 18 kb)

Additional file 5: Co-expression network of R1 vs Con. The DElncRNA-DEmRNA co-expression network of R1 vs Con. Rectangle nodes represented lncRNAs and the circulars represented mRNAs. The lines between nodes represented interactions between two genes. Red nodes meant the upregulated genes while green nodes meant the downregulated genes. Degree was judged by the number of links one node has with other nodes. (TIF 1912 kb)

Additional file 6: Co-expression network of R2 vs Con. The DElncRNA-DEmRNA co-expression network of R2 vs Con. Rectangle nodes represented lncRNAs and the circulars represented mRNAs. The lines between nodes represented interactions between two genes. Red nodes meant the upregulated genes while green nodes meant the downregulated genes. Degree was judged by the number of links one node has with other nodes. (TIF 1677 kb)

## Abbreviations

CAM: Cell adhesion molecules
cDNA: Complementary DNA
DElncRNAs: Differentially expressed lncRNAs
DEmRNAs: Differentially expressed mRNAs
DMEM: Dulbecco’s modified Eagle’s medium
ECM: Extracellular matrix
FBS: Fetal bovine serum
FC: Foldchange
GO: Gene ontology
HCC: Hepatocellular carcinoma
KEGG: Kyoto Encyclopedia of Genes and Genomes
lncRNAs: Long non-coding RNAs
miRNA: MicroRAN
mRNA: Message RNA
PPAR: Peroxisome proliferator-activated receptors
PVDF: Polyvinylidene difluoride
qRT-PCR: Quantitative real-time PCR
ROCK: Rho-associated coiled-coil containing kinases
ROS: Reactive oxygen species
VEGF: Vascular endothelial growth factor
