# Supplementary figures and images for: Overlapping and unique roles played by ROCK1 and 2 in the modulation of coding and long noncoding RNA expression
Source: BMC Genomics. 2019 May 22;20:409. doi: 10.1186/s12864-019-5715-0 (PMC6532151; doi:10.1186/s12864-019-5715-0)

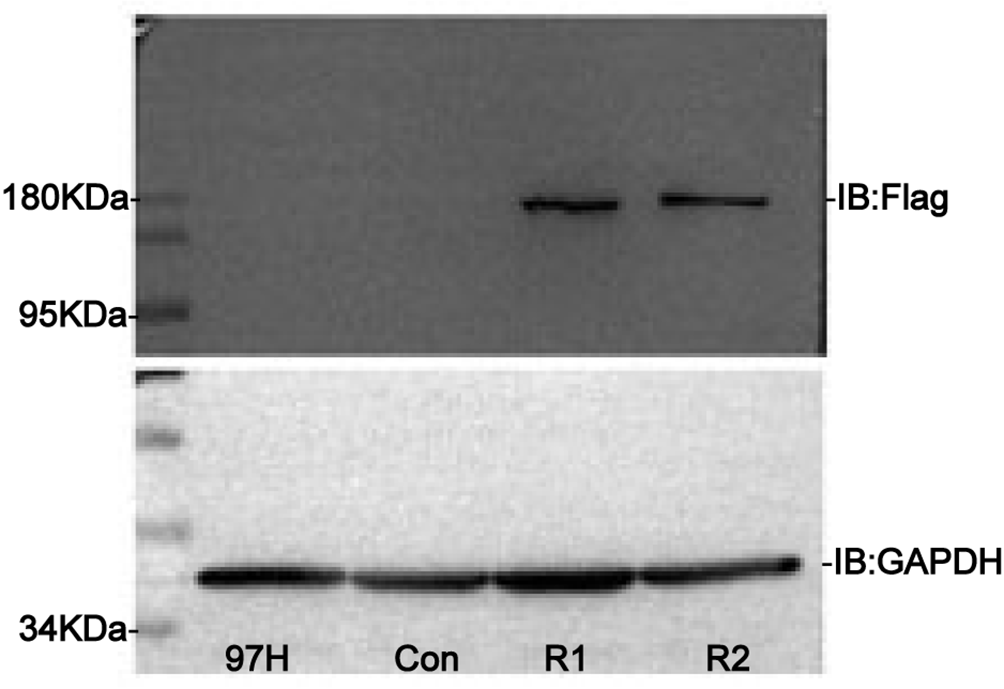

Supplement: Supplementary file 1 — Establishment of ROCK1 & 2 stably expressing cells. Western blot was performed to confirm the protein expression of ROCK1 & 2 in MHCC-97H cell lines, using GAPDH as internal reference. Left to right – MHCC-97H (97H), MHCC-97H-Con (Con), MHCC-97H-ROCK1 (R1) and MHCC-97H-ROCK2 (R2). (TIF 3040 kb) [file 12864_2019_5715_MOESM1_ESM.tif]

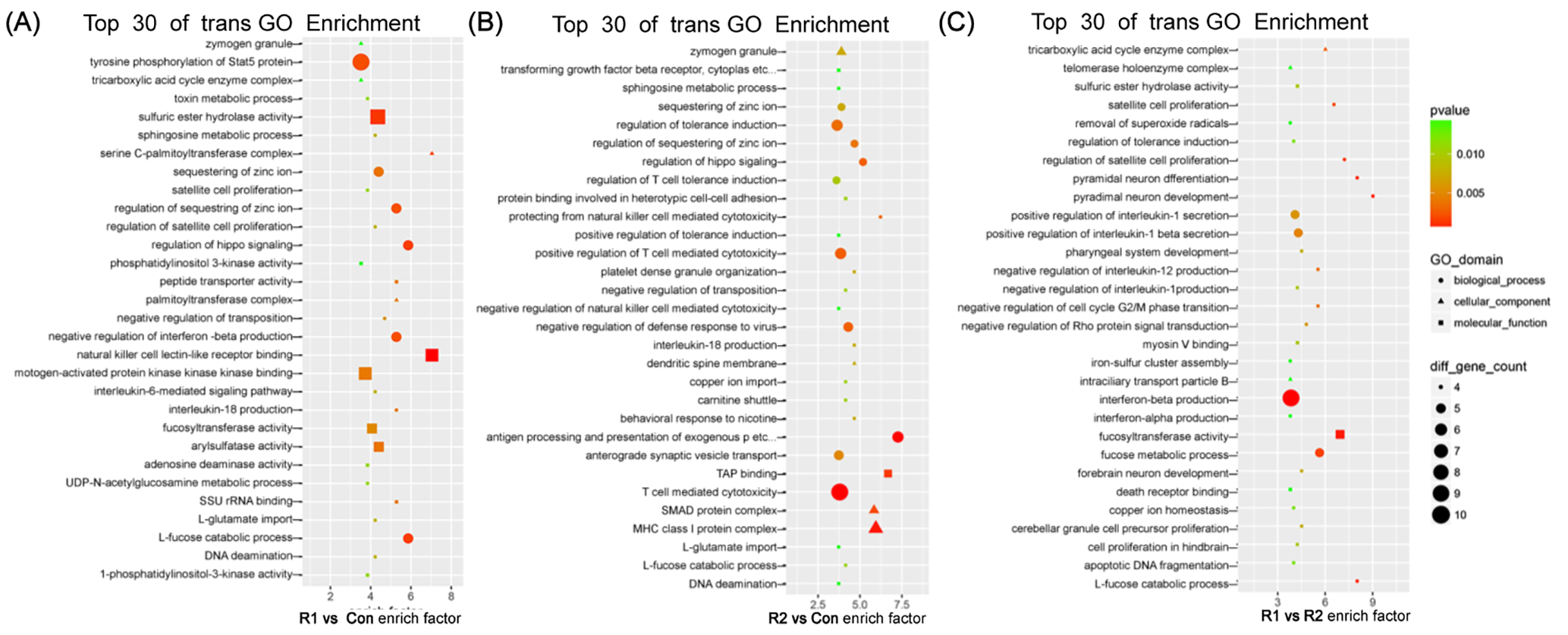

Supplement: Supplementary file 2 — GO enrichment analysis of trans prediction target. The top30 GO enrichment terms of trans prediction target genes of DElncRNAs were listed. The left one represented R1 vs Con, the next represented R2 vs Con and the last one represented R1 vs R2. The bubble charts illustrated the GO terms in which trans target genes of DElncRNAs enriched. The enrich factor was calculated by (number of different genes in a term/total number of different genes in a term) /(total number of genes in a term/total number of genes in a database). P < 0.05 was considered statistically significant. (TIF 6686 kb) [file 12864_2019_5715_MOESM2_ESM.tif]

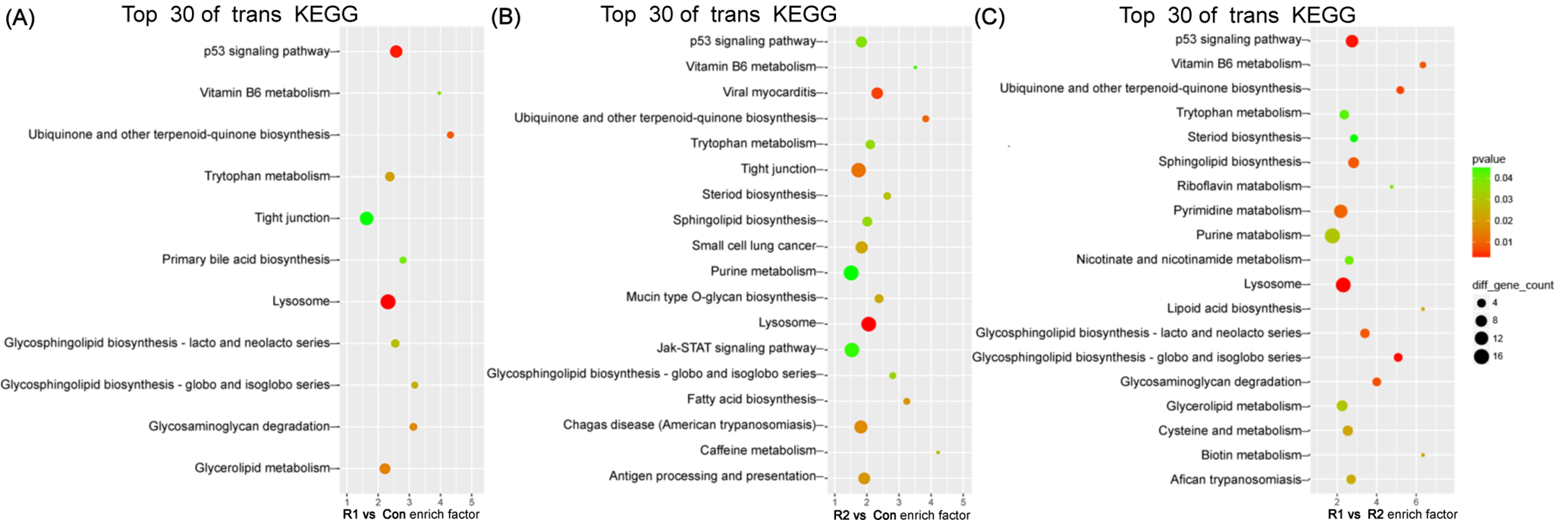

Supplement: Supplementary file 3 — KEGG analysis of trans prediction target. The top30 pathway enrichment terms of trans prediction target genes of DElncRNAs were listed. The left one represented R1 vs Con, the next represented R2 vs Con and the third one represented R1 vs R2. The bubble charts illustrated the KEGG terms in which trans target genes of DElncRNAs enriched. The enrich factor was calculated by (number of different genes in a term/total number of different genes in a term) /(total number of genes in a term/total number of genes in a database). P < 0.05 was considered statistically significant. (TIF 5262 kb) [file 12864_2019_5715_MOESM3_ESM.tif]

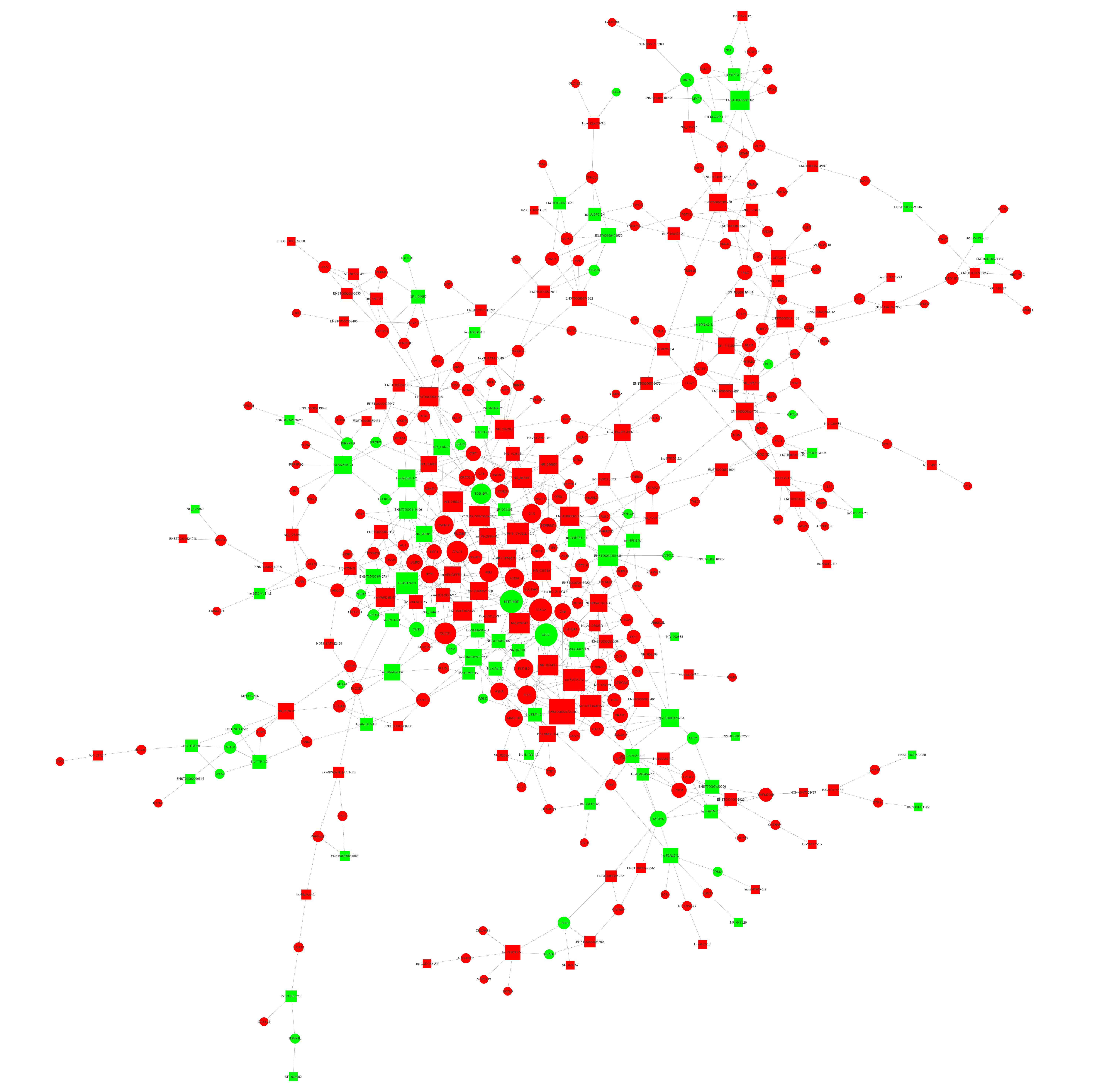

Supplement: Supplementary file 5 — Co-expression network of R1 vs Con. The DElncRNA-DEmRNA co-expression network of R1 vs Con. Rectangle nodes represented lncRNAs and the circulars represented mRNAs. The lines between nodes represented interactions between two genes. Red nodes meant the upregulated genes while green nodes meant the downregulated genes. Degree was judged by the number of links one node has with other nodes. (TIF 1912 kb) [file 12864_2019_5715_MOESM5_ESM.tif]

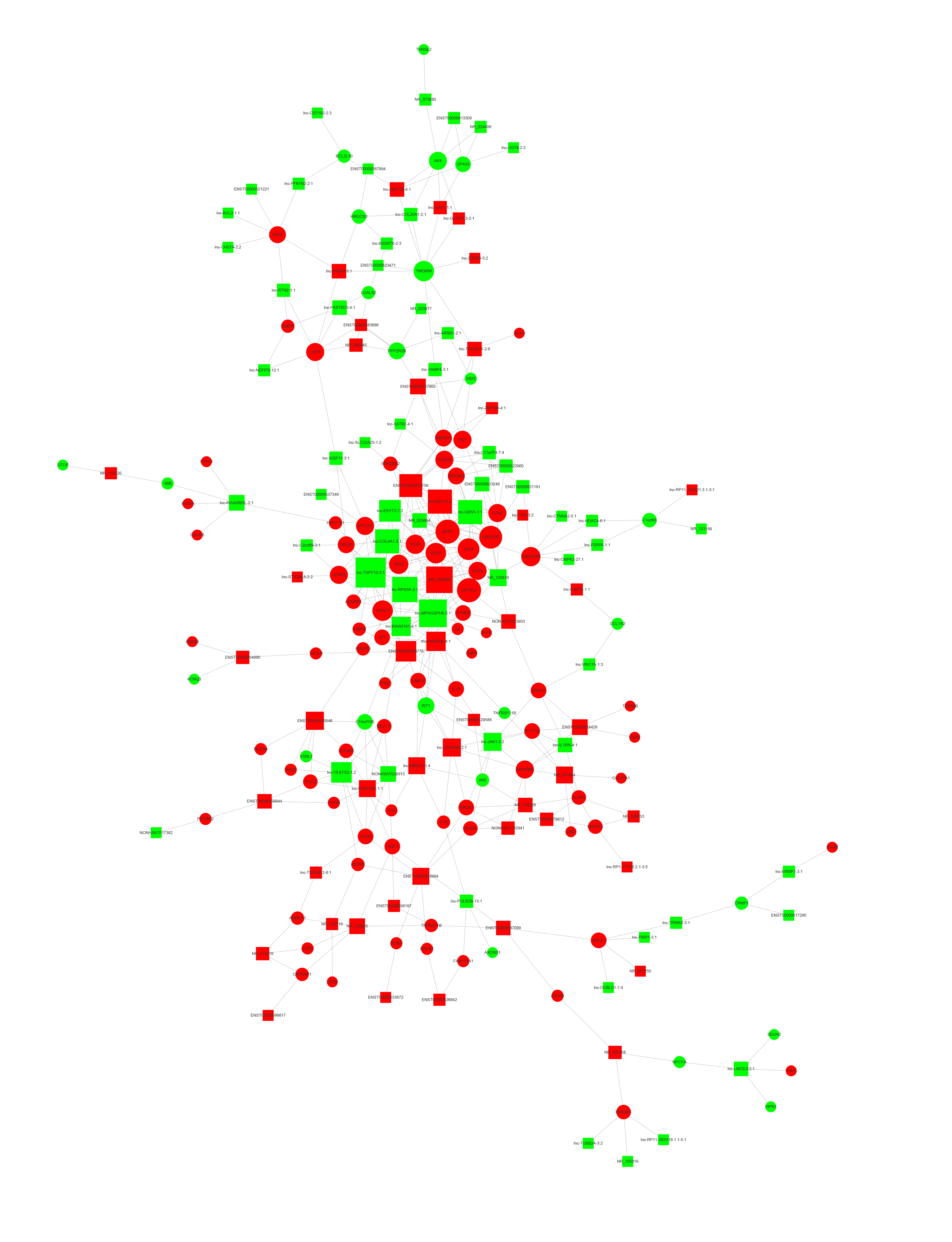

Supplement: Supplementary file 6 — Co-expression network of R2 vs Con. The DElncRNA-DEmRNA co-expression network of R2 vs Con. Rectangle nodes represented lncRNAs and the circulars represented mRNAs. The lines between nodes represented interactions between two genes. Red nodes meant the upregulated genes while green nodes meant the downregulated genes. Degree was judged by the number of links one node has with other nodes. (TIF 1677 kb) [file 12864_2019_5715_MOESM6_ESM.tif]
