# Supplementary material for: Overlapping and unique roles played by ROCK1 and 2 in the modulation of coding and long noncoding RNA expression
Source: BMC Genomics. 2019 May 22;20:409. doi: 10.1186/s12864-019-5715-0 (PMC6532151; doi:10.1186/s12864-019-5715-0)
Supplement: Supplementary file 4 — Method and Results of Reactome pathway analysis. The main functional implication of the DEmRNAs and DElncRNAs by Reactome analysis was listed, which included comparison between 3 groups: R1 vs Con, R2 vs Con and R1 vs R2. (DOCX 18 kb) [file 12864_2019_5715_MOESM4_ESM.docx]

**Supporting Materials**

**Title**

**Overlapping and unique roles played by ROCK1 & 2 in modulating coding and long non-coding RNA expression**

**Author information**

HeMing Zhou: zhmmango@outlook.com

JiGang Zhang: grissomzhang@hotmail.com

Xue Zhang: zhang52160@163.com

GuoRong Fan: fanguorong@sjtu.edu.cn

GaoLin Liu: gaolinliu@aliyun.com

Qin Li: liqin0626@hotmail.com

**Methods**

**Reactome pathway analysis**

Since GO gives a bit vague classification, another enrichment tool Reactome was used for further verification of results. The unique ID of MHCC-97H-ROCK1 vs MHCC-97H-Con is MjAxODEyMjEwMjE0MzVfMzAxOA%3D%3D for DEmRNAs, MjAxODEyMjQwNjE1MDBfMzU1OQ%3D%3D for DElncRNAs cis-target prediction, MjAxODEyMjQwNjI1MThfMzU2NQ%3D%3D for DElncRNAs trans-target prediction; ID of MHCC-97H-ROCK2 vs MHCC-97H-Con is MjAxODEyMjEwMjI5NDVfMzAyMw%3D%3D for DEmRNAs, MjAxODEyMjQwNjIwMDZfMzU2MQ%3D%3D for DElncRNAs cis-target prediction, MjAxODEyMjQwNjM0MTJfMzU2Ng%3D%3D for DElncRNAs trans-target prediction; and the ID of MHCC-97H-ROCK1 vs MHCC-97H-ROCK2 is MjAxODEyMjEwMjMyNTRfMzAyNQ%3D%3D for DEmRNAs, MjAxODEyMjQwNjIzMDdfMzU2Mw%3D%3D for DElncRNAs cis-target prediction, MjAxODEyMjQwNjQxMjNfMzU2Nw%3D%3D for DElncRNAs trans-target prediction.

**Results**

**Reactome analysis of differentially expressed mRNAs**

The functional implication of the DEmRNAs was investigated by Reactome. Based on the Reactome analysis, comparing with the MHCC-97H-ROCK1 cells, the DEmRNAs were especially enriched in following annotations: Endosomal/Vacuolar pathway, Integrin cell surface interactions, Activation of C3 and C5, Collagen degradation, RUNX2 regulates genes involved in cell migration, Assembly of collagen fibrils and other multimeric structures, ECM proteoglycans, Antigen Presentation: Folding, assembly and peptide loading of classⅠMHC, Post-translational protein phosphorylation, Transcriptional activation of p53 responsive genes, Transcriptional activation of cell inhibitor p21, TFAP2 (AP-2) family regulates transcription of cell cycle factors, Degradation of extracellular matrix, Regulation of Insulin-like Growth Factor (IGF) transport and uptake by Insulin-like Growth Factor Binding Proteins (IGFBPs), Extracellular matrix organization, Interleukin-4 and Interleukin-13 signaling, RNA PolymeraseⅠPromoter Opening, Collagen chain trimerization, NCAM1 interactions, Defective CHEB3 causes SEDCJD, CREB3 factors activate genes, Senescence-Associated Secretory Phenotype (SASP), SIRT1 negatively regulates rRNA expression and Activation of Matrix Metalloproteinases. To sum up, DEmRNAs affected cell migration, assembly and organization of ECM, p53 signaling pathway which regulated apoptosis and senescence, regulation of gene expression and so on. All the above was consistent with GO and KEGG analysis.

In a like mean, by Reactome analysis, when compared with MHCC-97H-Con, the unique DEmRNAs in MHCC-97H-ROCK2 were involved in Interleukin-10 signaling, Formation of the cornified envelope, Activation of C3 and C5, TypeⅠhemidesmosome assembly, Regulation of Insulin-like Growth Factor (IGF) transport and uptake by Insulin-like Growth Factor Binding Proteins (IGFBPs), Keratinization, Post-translational proteins phosphorylation, Degradation of the extracellular matrix, Ethanol oxidation, Defective CHST3 causes SEDCJD, Integrin cell surface interactions, Interleukin-4 and Interleukin-13 signaling, Cell junction organization, Chemokine receptors bind chemokines, Collagen degradation, Peptide ligand-binding receptors, Anchoring fibril formation, Alternative complement activation, L1CAM interactions, Voltage gated Postassium channels, Collagen chain trimerization, TNFs bind their physiological receptors, ECM proteoglycans, Regulation of Complement cascade and Extracellular matrix organization. Obviously, several immunomodulation signaling pathways and ECM organization were highlighted, so the EDmRNAs in this contrast clustered in inflammatory response, which was in keeping with GO and KEGG.

Furthermore, the MHCC-97H-ROCK1 and MHCC-97H-ROCK2 cells were contrasted. The DEmRNAs were clustered in Ethanol oxidation, Glucuronidation, Regulation of Insulin-like Growth Factor (IGF) transport and uptake by Insulin-like Growth Factor Binding Proteins (IGFBPs), RUNX1 regulates transcription of genes involved in differentiation of myeloid cells, Calcitonin-like ligand receptors, Insulin-like Growth Factor-2 mRNA Binding Proteins (IGF2BPs/IPs/VICKZs) bind RNA, Post-translational protein phosphorylation, O-linked glycosylation of mucins, Interleukin-35 signaling, Interleukin-10 signaling, Reactions specific to the hybrid N-glycan synthesis pathway, Transport of glycerol from adipocytes to the liver by Aquaporins, Defective SLCO1B1 causes hyperbilirubinemia, Rotor type (HBLRR), Defective SLCO1B3 causes hyperbilirubinemia, Rotor type (HBLRR), Glycogen storage disease type0 (liver GYS2), Phenylketonuria, Glycogen storage disease type Ⅳ (GBE1), Termination of O-glycan biosynthesis, Activation of BMF and translocation to mitochondria, PhaseⅡ-Conjugation of compounds, RAB geranylgeranylation, Interleukin-7 signaling, Intrinsic Pathway of Fibrin Clot Formation, ATF4 activates genes, HCN channels. It’s not hard to find that DEmRNAs here focused on metabolic disorder. It’s also worth mentioning that the results of Reactome emphasized DEmRNAs in this comparison involved in several interleukin-associated signaling pathways, which distinguished with GO and KEGG.

**Reactome enrichment analysis of DElncRNAs prediction targets**

Compared with MHCC-97H-ROCK1 and MHCC-97H-Con, cis target Reactome was enriched in Insulin-like Growth Factor-2 mRNA Binding Proteins (IGF2BPs/IMPs/VICKZs) bind RNA, Butyrophilin (BTN) family interactions, POU5F1 (OCT4), SOX2, NANOG repress genes related to differentiation, RUNX3 regulates RUNX1-mediated transcription, Integrin cell surface interactions, TP53 Regulated Transcription of Genes Involved in Cytochrome C Release, RUNX1 regulates genes involved in megakaryocyte differentiation and platelet function, FOXO-mediated transcription of cell cycle genes, Smooth Muscle Contraction, Activation of C3 and C5, SUMOylation of transcription cofactors, Laminin interactions, Uptake and actions of bacterial toxins, N-Glycan antennae elongation, Non-integrin membrane-ECM interactions, Phase 3-rapid repolarization, Ca2+ activated K+ channels, CHL1 interactions, Cargo concentration in the ER, TGFBR2 MSI Frameshift Mutants in Cancer, Activation and oligomerization of BAK protein, Activation, translocation and oligomerization of BAX, Formation of editosomes by ADAR proteins, Signaling by MET, Clearance of Nuclear Envelope Membranes from Chromatin. The results centered on apoptosis and proliferation, gene expression regulation and immune response.

While the trans target Reactome focused on TRAIL signaling, The activation of arylsulfatases, Zinc efflux and compartmentalization by the SLC30 family, Regulation of necroptotic cell death, Transcriptional regulation of pluripotent stem cells, Josephin domain DUBs, CASP8 activity is inhibited, Interleukin-6 family signaling, SMAD4 MH2 Domain Mutants in Cancer, Loss of Function of SMAD4 in Cancer, Transcriptional Regulation by E2F6, Regulation by c-FLIP, Dimerization of procaspase-8, PTK6 Activates STAT3, SMAC (DLABLO)-mediated dissociation of IAP: caspase complexes, POU5F1 (OCT4), SOX2, NANOG activate genes related to proliferation, Butyrophilin (BTN) family interactions, SMAC, XIAP-regulated apoptotic response, Activation of caspases through apoptosome-mediated cleavage, Regulated Necrosis, RIPK1-mediated regulated necrosis, HDR through Single Strand Annealing (SSA), Activation, myristolyation of BID and translocation to mitochondria, Formation of apoptosome. The pathways concentrated on immune response and apoptosis and proliferation.

In contrast to MHCC-97H-ROCK2 and MHCC-97H-Con, the cis target Reactome focused on Endosomal/Vascuolar pathway, Antigen Presentation: Folding, assembly and peptide loading of classⅠMHC, ER-Phagosome pathway, Interferon alpha/beta signaling, Antigen processing-Cross presentation, RHO GTPases activate IQGAPs, Interferon gamma signaling, Regulation of gene expression by Hypoxia-inducible Factor, Immunoregulatory interactions between a Lymphoid and a non-Lymphoid cell, POU5F1 (OCT4), SOX2, NANOG repress genes related to differentiation, Activation of PUMA and translocation to mitochondria, Ca2+ activated K+ channels, Dopamine Neurotransmitter Release Cycle, Defective B3GALTL causes Peters-plus syndrome (Pps), ClassⅠMHC mediated antigen processing & presentation, Diseases associated with O-glycosylation of proteins, ECM proteoglycans, Long-term potentiation, Cell-cell junction organization, Cell junction organization, Microtubule-dependent trafficking of connexons from Golgi to the plasma membrane, Tachykinin receptors bind tachykinins. The output oriented cell adhesion, cell junction and immune response.

While the trans target Reactome was observed in Endosomal/Vacuolar pathway, Antigen Presentation: Folding, assembly and peptide loading of classⅠMHC, Interferon alpha/beta signaling, ER-Phagosome pathway, Interferon gamma signaling, Antigen processing-Cross presentation, Interferon Signaling, Immunoregulatory interactions between a Lymphoid and a non-Lymphoid cell, ClassⅠMHC mediated antigen processing & presentation, TRAIL signaling, TP53 Regulates Transcription of Caspase Activators and Caspases, Zinc efflux and compartmentalization by the SLC30 family, The activation of arylsulfatases, SMAD4 MH2 Domain Mutants in Cancer, Loss of Function of SMAD4 in Cancer, RNA PolymeraseⅠTranscription Termination, Signaling by FGFR2 Ⅲa TM, TP53 Regulates Transcription of DNA Repair Genes, SMAC (DIABLO) binds to IAPs, SMAC (DIABLO)-mediated dissociation of IAP: caspase complexes, Activation of gene expression by SREBF (SREBP), RNA PolymeraseⅠPromoter Escape, Cytokine Signaling in Immune system, Transcriptional Regulation by E2F6. The results focused on immune response and gene expression regulation.

Furthermore, cis target genes and trans target genes were subjected to Reactome between MHCC-97H-ROCK1 and MHCC-97H-ROCK2. The DElncRNAs target prediction in MHCC-97H-ROCK1 indicated the following: the cis target Reactome enriched in CHL1 interactions, CASP8 activity is inhibited, Caspase activation via Death Receptors in the presence of ligand, Regulation of necroptotic cell death, Activation and oligomerization of BAK protein, Formation of editosomes by ADAR proteins, Activation, translocation and oligomerization of BAX, RAB geranylgeranylation, ARL13B-mediated ciliary trafficking of INPP5E, RIPK1-mediated regulated necrosis, Regulated Necrosis, Caspase activation via extrinsic apoptotic signaling pathway, Activation, myristolyation of BID and translocation to mitochondria, Antagonism of Activin by Follistatin, Defective SLC7A7 causes lysinuric protein intolerance (LPI), Defective CP causes aceruloplasminemia (ACERULOP), mRNA Editing: A to I Conversion, C6 deamination of adenosine, TP53 Regulates Transcription of Genes Involved in Cytochrome C Release, GABA synthesis, release, reuptake and degradation, DNA Damage Recognition in GG-NER, Death Receptor Signaling, Activation of AMPA receptors, RUNX2 regulates chondrocyte maturation, Interleukin-1 processing. Apoptosis, necroptosis and RNA editing was emphasized here.

While the trans target Reactome focused on TRAIL signaling, The activation of arylsulfatases, CASP8 activity is inhibited, Regulation of necroptotic cell death, Activation, myristolyation of BID and translocation to mitochondria, Signaling by FGFR2 Ⅲa TM, Phospholipase C-mediated cascade; FGFR2, RORA activates gene expression, ROBO receptors bind AKAP5, Regulation by c-FLIP, Dimerization of procaspase-8, Apoptotic cleavage of cell adhesion proteins, IRAK4 deficiency (TLR5), FGFR2 ligand binding and activation, Interleukin-21 signaling, Apoptotic execution phase, RIPK1-mediated regulated necrosis, Regulated Necrosis, Caspase activation via Death Receptors in the presence of ligand, Pyrophosphate hydrolysis, Transcriptional Regulation by E2F6, Josephin domain DUBs, Activation of DNA fragmentation factor, Apoptosis induced DNA fragmentation, Inhibition of replication initiation of damaged DNA by RB1/E2F1. The results clustered on apoptosis, immune-associated signaling and DNA damage.
